# Phytochemical Characterization and Antioxidant Mechanisms of Amazonian Tucumã (*Astrocaryum vulgare*) and Uxi (*Endopleura uchi*) Pulps Support Their Potential as Natural Food Preservatives

**DOI:** 10.1111/1750-3841.71476

**Published:** 2026-09-16

**Authors:** Naikita Suellen da Silva e Silva, Thaís Oliveira Freitas, Ana Paula Barros Soares, Bruna Sumaya Dias Pinheiro, Saulo Victor e Silva, Anelise Christ‐Ribeiro, Jefferson Romáryo Duarte da Luz, Gabriel Araujo‐Silva

**Affiliations:** ^1^ Postgraduate Program in Pharmaceutical Sciences, PPgCF Federal University of Amapá (UNIFAP) Macapá Brazil; ^2^ Organic Chemistry and Biochemistry Laboratory Amapá State University (UEAP) Macapá Brazil; ^3^ Department of Food Science and Nutrition University of Antofagasta Antofagasta Chile; ^4^ School of Chemistry and Food Federal University of Rio Grande (FURG) Rio Grande Brazil; ^5^ College of Biological Sciences Federal University of Amapá (UNIFAP) Oiapoque Brazil

**Keywords:** Amazonian fruits, antioxidant activity, *Astrocaryum vulgare*, bioactive compounds, *Endopleura uchi*, food preservation, natural preservatives

## Abstract

The growing demand for clean‐label foods has intensified the search for natural alternatives to synthetic preservatives. Amazonian fruits are promising sources of bioactive compounds with antioxidant properties that may improve food preservation. This study evaluated the phytochemical composition, fatty acid profile, and antioxidant potential of pulps from tucumã (*Astrocaryum vulgare*) and uxi (*Endopleura uchi*) to assess their suitability as natural food preservatives. Pulp extracts were characterized by total phenolic and flavonoid contents, fatty acid composition, and high‐performance liquid chromatography coupled with diode‐array detection (HPLC‐DAD). Antioxidant activity was evaluated using ferric reducing antioxidant power (FRAP), total antioxidant capacity (TAC), DPPH radical scavenging, and thiobarbituric acid reactive substances (TBARS) assays. Tucumã contained significantly higher levels of total phenolics (36.21 ± 1.99 mg GAE/g extract) and flavonoids (16.16 ± 0.21 mg QE/g extract) than uxi (31.56 ± 0.85 mg GAE/g extract and 5.68 ± 0.45 mg QE/g extract, respectively). Fatty acid analysis revealed distinct lipid profiles, with tucumã predominated by linoleic acid and uxi by oleic acid. HPLC‐DAD identified gallic acid as the major phenolic compound in uxi, whereas tucumã exhibited a more diverse phytochemical profile, including citric acid and quercetin derivatives. Despite its lower phenolic content, uxi showed significantly higher FRAP values and greater DPPH radical scavenging activity at concentrations ≥ 100 mg/L, whereas TAC and EC_50_ values did not differ significantly between the extracts. Both fruits effectively inhibited lipid peroxidation. These findings demonstrate that antioxidant performance depends on phytochemical composition rather than solely on total phenolic content, highlighting tucumã and uxi as promising natural antioxidants for food preservation and functional food applications.

## 1 Introduction

The increasing consumer demand for minimally processed and clean‐label foods has intensified the search for natural alternatives to synthetic food additives. Among these additives, synthetic antioxidants such as butylated hydroxytoluene (BHT) and butylated hydroxyanisole (BHA) have been widely employed to delay lipid oxidation and extend the shelf life of food products (Parveen et al. 2025; Maddaloni et al. 2025). Nevertheless, growing concerns regarding the long‐term consumption of synthetic preservatives, together with consumer preference for naturally derived ingredients, have encouraged the development of safer and more sustainable preservation strategies. This trend is consistent with modern food‐development approaches that increasingly integrate food processing, preventive health, scientific validation, and consumer demand for naturally derived functional ingredients (Sun‐Waterhouse et al. 2024). Consequently, plant‐derived bioactive compounds have emerged as promising candidates for replacing synthetic antioxidants in food systems (Antony and Narayanaswamy 2026).

Natural antioxidants obtained from fruits, vegetables, herbs, and other botanical sources have attracted considerable attention because of their ability to scavenge reactive oxygen species, donate electrons or hydrogen atoms, chelate transition metals, and inhibit lipid oxidation (Parveen et al. 2025; Gulcin 2025). Among these compounds, phenolic acids, flavonoids, carotenoids, vitamins, and unsaturated fatty acids have been extensively investigated due to their capacity to improve oxidative stability while simultaneously enhancing the nutritional value of foods (Debri et al. 2026). However, growing evidence indicates that antioxidant activity depends not only on the total concentration of bioactive compounds but also on their chemical structure, redox properties, and interactions within complex food matrices (Arias et al. 2022; Gulcin 2025; Edkaidek et al. 2026). Therefore, comprehensive phytochemical characterization is essential to understand the mechanisms underlying the antioxidant performance of plant extracts.

The Amazon rainforest is recognized as one of the world's richest reservoirs of biodiversity and harbors numerous fruit species with remarkable nutritional and functional properties (Araujo et al. 2021). Amazonian fruits have gained increasing scientific attention because they combine high concentrations of bioactive phytochemicals with unique lipid compositions rich in nutritionally valuable unsaturated fatty acids (Santos et al. 2015; Araujo et al. 2021; Araujo et al. 2023). These characteristics make them attractive candidates for replacing synthetic antioxidants in food preservation, particularly considering that lipid‐rich foods are highly susceptible to oxidative deterioration (Poljsak et al. 2021). In addition to extending shelf life, natural antioxidants from Amazonian fruits may contribute to the development of functional foods with added nutritional and health‐promoting properties (Araújo et al. 2023).

Among these native species, tucumã (*Astrocaryum vulgare*) and uxi (*Endopleura uchi*) stand out because of their chemical composition and traditional consumption throughout the Amazon region (Oliveira et al. 2021; Machado et al. 2022). Tucumã has been described as an important source of carotenoids, phenolic compounds, flavonoids, and unsaturated fatty acids, whereas uxi contains phenolic metabolites associated with antioxidant and anti‐inflammatory activities (Oliveira et al. 2021; Machado et al. 2022; Gualberto et al. 2025). Despite their recognized nutritional value, the phytochemical composition of these fruits differs substantially, suggesting that they may exhibit distinct antioxidant mechanisms and technological properties. Such differences are particularly relevant for food preservation, since antioxidant effectiveness depends on the ability of specific compounds to inhibit different oxidative pathways (Brito et al. 2026).

Although previous studies have investigated nutritional composition or biological activities of tucumã and uxi individually (Oliveira et al. 2021; Machado et al. 2022), comparative studies integrating phytochemical characterization, fatty acid profiling, chromatographic analysis, and complementary antioxidant assays remain scarce. Moreover, little is known about how differences in phytochemical composition influence distinct antioxidant mechanisms (Apak et al. 2016). This information is essential because the antioxidant performance of plant extracts cannot be explained solely by the total concentration of phenolic compounds, but also by the qualitative composition, molecular structure, redox properties, and interactions of their bioactive constituents (Shi et al. 2022; Cunha et al. 2026). Therefore, a direct comparison of these two Amazonian fruits using complementary analytical approaches may provide a more comprehensive understanding of their antioxidant behavior and their potential application as natural food preservatives.

Based on these considerations, we hypothesized that the distinct phytochemical compositions of tucumã and uxi would result in different antioxidant response patterns across assays based on electron transfer, radical scavenging, and inhibition of lipid peroxidation. We further hypothesized that differences in their fatty acid profiles would contribute to distinct oxidative behaviors, thereby influencing their suitability for different food preservation applications. Therefore, the present study aimed to comparatively characterize the phytochemical composition, fatty acid profile, chromatographic profile, and antioxidant mechanisms of lyophilized pulps from tucumã (*A. vulgare*) and uxi (*E. uchi*). Total phenolic and flavonoid contents, fatty acid composition, HPLC‐DAD chromatographic profiles, ferric reducing antioxidant power (FRAP), total antioxidant capacity (TAC), DPPH radical scavenging activity, and inhibition of lipid peroxidation (TBARS) were evaluated to determine how differences in chemical composition influence antioxidant mechanisms and to assess the potential of these Amazonian fruits as natural antioxidant ingredients for food preservation.

## 2 Material and Methods

### 2.1 Plant Material and Lyophilization

Ripe fruits of tucumã (*A. vulgare*) were collected in the district of São Joaquim do Pacuí, rural area of Macapá, Amapá State, Brazil (0.716692, −50.795263). Uxi fruits (*E. uchi*) were obtained from a local public market in Macapá, Amapá State, Brazil (0.023580, −51.077987). Access to the Brazilian genetic heritage was registered in the National System for the Management of Genetic Heritage and Associated Traditional Knowledge (SISGEN) under registration number A979AB2.

The fruits were washed under running water, manually pulped, and separated from seeds and residual materials. The edible portions were frozen in liquid nitrogen and subsequently freeze‐dried using a laboratory lyophilizer (JJ Científica, LJJ Series Freeze Dryer, São Paulo, Brazil) under vacuum (50–200 µHg) at −98°C. After lyophilization, the samples were stored in sealed polyethylene containers at −18°C until extraction.

Hydroalcoholic extracts were obtained by maceration of the lyophilized pulps in 70% ethanol (1:20, w/v) for 7 days at room temperature (25 ± 2°C) with occasional stirring. The extracts were filtered through qualitative filter paper, and the solvent was removed in a drying oven at 50°C until complete evaporation. The dried extracts were cooled in a desiccator and subsequently reconstituted in the appropriate solvent according to the requirements of each analytical assay. All chemical characterization and antioxidant analyses were performed using the hydroalcoholic extracts obtained from the lyophilized pulps.

### 2.2 Determination of Total Phenolic Content

The total phenolic content (TPC) of the hydroalcoholic extracts was determined using the Folin–Ciocalteu colorimetric method with minor modifications (Obeng et al. 2020). Briefly, 25 µL of each extract were mixed with 2.75 mL of Folin–Ciocalteu reagent (3%, v/v). After 5 min of reaction at room temperature, 25 µL of sodium carbonate solution (10%, w/v) were added, and the mixture was incubated in the dark for 60 min.

Absorbance was measured at 765 nm using a UV–vis spectrophotometer (BEL UV‐M51, Bel Engineering, Italy). A reagent blank containing methanol instead of extract was prepared under identical conditions. Quantification was performed using a gallic acid standard curve (20–100 mg/L), and the results were expressed as milligrams of gallic acid equivalents per gram of dry extract (mg GAE/g extract). All analyses were carried out in triplicate.

### 2.3 Determination of Total Flavonoid Content

The total flavonoid content (TFC) of the hydroalcoholic extracts was determined using the aluminum chloride colorimetric method described by Morais et al. (2022), with minor modifications. Briefly, the extracts were prepared at a concentration of 5 mg/mL and mixed with an equal volume of 2% (w/v) aluminum chloride (AlCl_3_) solution. Subsequently, 1 mL of sodium hydroxide (NaOH, 1 M) was added, and the reaction mixture was homogenized and incubated at room temperature for 30 min to allow chromophore formation.

Absorbance was measured at 425 nm using a UV–vis spectrophotometer (BEL UV‐M51, Bel Engineering, Italy). Quantification was performed using a quercetin calibration curve (5–100 µg/mL), and the results were expressed as milligrams of quercetin equivalents per gram of dry extract (mg QE/g extract). All measurements were carried out in triplicate.

### 2.4 Fatty Acid Composition Analysis

Total lipids were extracted from the lyophilized pulps according to the method described by Folch et al. (1957). The lipid fraction was isolated using an organic solvent extraction system, and the recovered oils were subjected to methyl ester derivatization following the procedure described by Hartman and Lago (1973).

Approximately 30 mg of extracted oil were mixed with 500 µL of potassium hydroxide solution (KOH, 0.1 mol/L), vortexed for 1 min, and incubated in a water bath at 60°C for 1.5 h. Subsequently, 1.5 mL of sulfuric acid solution (H_2_SO_4_, 1 mol/L) were added, followed by a second incubation at 60°C for 1.5 h. After cooling, 2 mL of HPLC‐grade n‐hexane were added, and the mixture was vortexed for 1 min. After phase separation, the upper organic phase containing fatty acid methyl esters (FAMEs) was collected for chromatographic analysis.

FAMEs were analyzed using a gas chromatograph equipped with a flame ionization detector (GC‐FID; GC‐2010 Plus, Shimadzu, Kyoto, Japan), a split/splitless injector, and a Zebron ZB‐FAME capillary column (20 m × 0.18 mm i.d., 0.15 µm film thickness). The injector and detector temperatures were set at 250°C and 260°C, respectively. Nitrogen (N_2_) was used as the carrier gas.

The oven temperature program was as follows: initial temperature of 80°C held for 1.5 min, increased to 160°C at 40°C/min, then increased to 185°C at 5°C/min, and finally increased to 260°C at 30°C/min, with a total run time of 11 min. Fatty acids were identified by comparing the retention times (RTs) of sample peaks with those of a commercial FAME standard mixture (Supelco 37 Component FAME Mix, Sigma‐Aldrich, St. Louis, MO, USA). Quantification was performed by peak area normalization, and results were expressed as relative percentages of total fatty acids.

### 2.5 HPLC‐DAD Analysis

The chromatographic profile of the hydroalcoholic extracts was evaluated by high‐performance liquid chromatography coupled with a diode‐array detector (HPLC‐DAD). Analyses were performed using an LC‐2030C 3D Plus HPLC system equipped with a photodiode array detector (Shimadzu Corporation, Kyoto, Japan) and a Kinetex C18 column (100 mm × 4.6 mm, 2.6 µm particle size; Phenomenex, Torrance, CA, USA), maintained at 40°C.

The mobile phase consisted of (A) water containing 0.1% formic acid, (B) acetonitrile, (C) 10% isopropanol, and (D) distilled water. Separation was achieved using a previously established gradient elution program adapted from Carvalho et al. (2023). Chromatograms were monitored at 254 and 350 nm, allowing the detection of phenolic compounds and flavonoid‐related metabolites.

Compounds were tentatively assigned based on comparisons of chromatographic RTs and UV‐Vis absorption spectra with available reference standards and published data. Authentic standards were not co‐injected for all tentatively assigned compounds. Therefore, the chromatographic assignments should be regarded as tentative rather than unequivocal identifications. Quantitative results were considered semi‐quantitative and expressed as equivalents of the respective reference standards based on their calibration curves, rather than as absolute concentrations of definitively identified compounds.

### 2.6 DPPH Radical Scavenging Assay

The free radical scavenging activity of the hydroalcoholic extracts was evaluated using the 2,2‐diphenyl‐1‐picrylhydrazyl (DPPH•) assay according to Brand‐Williams (1995), with minor modifications. Briefly, 100 µL of each extract solution (5 mg/mL) were mixed with 3.9 mL of a DPPH methanolic solution (60 µM). The reaction mixtures were incubated in the dark at 20°C for 30 min.

Absorbance was measured at 512 nm using a UV–vis spectrophotometer (BEL UV‐M51, Bel Engineering, Italy). Radical scavenging activity was calculated as the percentage of DPPH inhibition according to Equation (1):

$$\%\mathrm{SR}\;=100-\left[\frac{\left(\mathrm{Abs}\;\mathrm{control}-\mathrm{Abs}\;\mathrm{sample}\right)}{\left(\mathrm{Abs}\;\mathrm{control}-\mathrm{Abs}\;\mathrm{blank}\right)}\;\times 100\right]$$

The antioxidant capacity of the extracts was compared with BHT used as a positive control. The effective concentration required to scavenge 50% of DPPH radicals (EC_50_) was determined by linear regression analysis of inhibition percentage versus extract concentration. Lower EC_50_ values were considered indicative of higher antioxidant activity. All measurements were performed in triplicate.

### 2.7 Ferric Reducing Antioxidant Power

The ferric reducing antioxidant power (FRAP) assay was performed according to the method described by Benzie and Strain (1996). Briefly, 30 µL of each extract solution (2.000 µg/mL) were added to 900 µL of freshly prepared FRAP reagent.

The FRAP reagent consisted of acetate buffer (0.3 M, pH 3.6), 2,4,6‐tripyridyl‐s‐triazine (TPTZ) solution, and ferric chloride hexahydrate (FeCl_3_·6H_2_O) solution mixed in the ratio of 10:1:1 (v/v/v). After incubation under the reaction conditions, absorbance was measured at 593 nm using a UV‐Vis spectrophotometer (BEL UV‐M51, Bel Engineering, Italy).

Quantification was performed using a calibration curve prepared with ferrous sulfate heptahydrate (FeSO_4_·7H_2_O) at concentrations ranging from 0.0833 to 0.5 mM. Results were expressed as millimoles of Fe^2+^ equivalents per gram of dry extract (mmol Fe^2+^/g extract). All measurements were carried out in triplicate.

### 2.8 Total Antioxidant Capacity

The TAC of the hydroalcoholic extracts was determined using the phosphomolybdenum method described by Prieto et al. (1999). Briefly, 300 µL of each extract solution (200 µg/mL) were mixed with 2.7 mL of phosphomolybdenum reagent solution containing 0.03 M ammonium molybdate, 0.1 M sodium phosphate, and 3 M sulfuric acid.

The reaction mixtures were incubated at 95°C for 90 min in a water bath and subsequently cooled to room temperature. Absorbance was measured at 695 nm using a UV–vis spectrophotometer (BEL UV‐M51, Bel Engineering, Italy).

Quantification was performed using an ascorbic acid calibration curve (12–240 µg/mL), and the results were expressed as grams of ascorbic acid equivalents per gram of dry extract (g AAE/g extract). All analyses were performed in triplicate.

### 2.9 Inhibition of Lipid Peroxidation (TBARS Assay)

The ability of the hydroalcoholic extracts to inhibit lipid peroxidation was evaluated using the thiobarbituric acid reactive substances (TBARS) assay, according to Melo et al. (2011), with minor modifications. Egg yolk homogenate (1%, w/v) prepared in phosphate‐buffered saline (PBS, 20 mM, pH 7.4) was used as the lipid‐rich substrate.

Briefly, 1.0 mL of the egg yolk emulsion was mixed with 100 µL of extract solutions at concentrations of 50, 500, and 5000 µg/mL. BHT and quercetin were used as positive controls. Lipid peroxidation was induced by adding 100 µL of 2,2′‐azobis(2‐amidinopropane) dihydrochloride (AAPH, 0.12 M), followed by incubation at 37°C for 30 min.

After incubation, 4.0 mL of thiobarbituric acid‐trichloroacetic acid reagent (TBA‐TCA; 0.73% thiobarbituric acid in 15% trichloroacetic acid) were added, and the mixtures were heated at 95°C for 45 min. The samples were then cooled in an ice bath and extracted with 3.0 mL of n‐butanol. Following centrifugation at 7500 rpm for 10 min, the absorbance of the organic phase was measured at 532 nm using a UV–vis spectrophotometer (BEL UV‐M51, Bel Engineering, Italy).

Positive controls consisted of samples containing AAPH without extract, whereas negative controls contained neither AAPH nor extract. The inhibitory effect of the extracts on lipid peroxidation was determined by comparison with the control groups, and results were expressed as TBARS equivalents per gram of extract. All experiments were performed in quintuplicate.

### 2.10 Statistical Analysis

Statistical analyses were performed using GraphPad Prism version 8.0 (GraphPad Software, San Diego, CA, USA). Data normality was assessed using the Shapiro–Wilk test, whereas homogeneity of variances was evaluated using the Brown–Forsythe or Bartlett's tests, as appropriate for each dataset. Data are presented as mean ± standard deviation (SD).

Comparisons between two independent groups were performed using unpaired Student's *t*‐test for TPC and TFC, and Welch's unpaired *t*‐test for FRAP and TAC, according to variance homogeneity. Fatty acid composition was compared using multiple unpaired Welch's *t*‐tests followed by false discovery rate (FDR) correction using the two‐stage step‐up procedure of Benjamini, Krieger, and Yekutieli (*Q* = 5%).

DPPH radical scavenging activity was analyzed by two‐way analysis of variance (ANOVA), considering sample type and extract concentration as fixed factors, followed by Sidak's multiple comparisons test. Differences among EC_50_ values were evaluated using one‐way ANOVA followed by Tukey's multiple comparisons test. Lipid peroxidation inhibition (TBARS) was analyzed using one‐way ANOVA followed by Tukey's multiple comparisons test. Differences were considered statistically significant at *p* < 0.05. Adjusted *p* values were reported for analyses involving multiple comparisons whenever applicable.

## 3 Results and Discussion

### 3.1 Phenolic Compounds and Flavonoids

Phenolic compounds and flavonoids are among the main phytochemical classes associated with the antioxidant properties of plant‐derived foods (Mutha et al. 2021). In the present study, both Amazonian fruits exhibited considerable concentrations of these bioactive compounds, supporting their potential use as natural antioxidant ingredients.

TPC differed significantly between the two fruit extracts. *A. vulgare* (tucumã) exhibited a higher phenolic content than *E. uchi* (uxi), with values of 36.21 ± 1.99 and 31.56 ± 0.85 mg gallic acid equivalents (GAE)/g extract, respectively (Student's unpaired *t*‐test, t[4] = 3.971, *p* = 0.0165). These results indicate that both fruits are relevant sources of phenolic compounds, which are recognized for their ability to donate electrons or hydrogen atoms, neutralize reactive oxygen species, and delay oxidative reactions in food and biological systems (Avila‐Sosa et al. 2019).

A more pronounced difference was observed for TFC. Tucumã presented 16.16 ± 0.21 mg quercetin equivalents (QE)/g extract, whereas uxi contained 5.68 ± 0.45 mg QE/g extract. This difference was highly significant (Student's unpaired *t*‐test, t[4] = 36.14, *p* < 0.0001), indicating that flavonoids represent a major fraction of the phenolic composition of tucumã. These compounds are widely recognized for their antioxidant activity, acting through radical scavenging, metal chelation, and interruption of oxidative chain reactions (Chandra et al. 2014).

The concentrations observed in both fruits are consistent with previous reports describing Amazonian species as important reservoirs of bioactive metabolites. Tucumã has been described as a source of phenolic compounds and carotenoids, while uxi contains phenolic constituents associated with antioxidant and anti‐inflammatory properties. Variations among studies may be explained by differences in environmental conditions, maturity stage, geographical origin, extraction procedures, and analytical methods (Machado et al. 2022; Daleaste et al. 2025).

From a technological perspective, the presence of substantial amounts of phenolic compounds and flavonoids is particularly relevant because these metabolites may contribute to oxidative stability and help delay lipid deterioration in food products (Rahman et al. 2021; Cunha et al. 2026). Therefore, the phytochemical composition observed in the present study reinforces the potential of tucumã and uxi as natural sources of antioxidant compounds for food preservation applications.

### 3.2 Fatty Acid Composition

The fatty acid composition of tucumã (*A. vulgare*) and uxi (*E. uchi*) is presented in Table 1. Comparison of the lipid profiles revealed marked differences between the two Amazonian fruits. After correction for multiple comparisons using the Benjamini–Krieger–Yekutieli false discovery rate (FDR; *Q* = 5%), 10 of the 12 comparable fatty acids differed significantly between species, demonstrating that tucumã and uxi possess distinct fatty acid signatures despite sharing the same Amazonian ecosystem.

**TABLE 1:** Comparative fatty acid composition of the pulps of *Astrocaryum vulgare* and *Endopleura uchi*.

Fatty acid	*Astrocaryum vulgare* (%)	*Endopleura uchi* (%)	Adjusted *q*‐value
C11:0	ND	0.43 ± 0.19	—
C12:0	0.02 ± 0.00	ND	—
C14:0	0.08 ± 0.00^a^	0.18 ± 0.06^b^	0.010
C16:0	28.63 ± 0.32^a^	18.32 ± 0.14^b^	< 0.000001
C16:1	0.25 ± 0.01^a^	0.15 ± 0.03^b^	0.0011
C17:0	0.09 ± 0.01^a^	0.22 ± 0.03^b^	0.0007
C17:1	0.05 ± 0.00	ND	—
C18:0	ND	8.21 ± 0.13	—
C18:1 T	2.03 ± 0.14	ND	—
C18:1 C	ND	58.85 ± 0.67	—
C18:2 T	64.03 ± 0.29^a^	0.31 ± 0.07^b^	< 0.000001
C18:2 C	2.73 ± 0.11^a^	6.24 ± 0.15^b^	0.000003
C18:3 C (9,12,15)	1.62 ± 0.08^a^	3.55 ± 0.14^b^	0.000014
C20:0	0.13 ± 0.01^a^	0.84 ± 0.19^b^	0.0008
C20:1	0.20 ± 0.00^a^	0.75 ± 0.17^b^	0.0010
C20:2	0.02 ± 0.00^a^	1.21 ± 0.33^b^	0.0009
C22:0	0.02 ± 0.00	0.22 ± 0.00	0.000005
C22:1	0.02 ± 0.01	ND	—
C22:2	0.01 ± 0.00^a^	0.36 ± 0.02^b^	—
C23:0	0.02 ± 0.00	ND	—
C24:0	0.05 ± 0.01^a^	0.15 ± 0.07^b^	0.016

*Note*: Data are expressed as mean ± standard deviation (SD) of triplicate analyses and reported as relative percentage (%). Different superscript letters within the same row indicate statistically significant differences between *Astrocaryum vulgare* and *Endopleura uchi*, as determined by Welch's unpaired *t*‐test followed by the Benjamini–Krieger–Yekutieli false discovery rate (FDR) correction (*Q* = 5%). ND indicates that the fatty acid was not detected under the analytical conditions employed. The symbol “—” in the adjusted *q*‐value column indicates that statistical comparison was not performed because the fatty acid was detected in only one species or because the test could not be computed (C22:0).

Tucumã exhibited a lipid profile predominantly composed of unsaturated fatty acids, with trans‐linoleic acid (C18:2T) representing the major constituent (64.03 ± 0.29%), followed by palmitic acid (C16:0; 28.63 ± 0.32%). Both fatty acids were present at significantly higher concentrations than those observed in uxi (*q* < 0.000001). Palmitoleic acid (C16:1) was also significantly more abundant in tucumã (*q* = 0.001133). In addition, lauric acid (C12:0), heptadecenoic acid (C17:1), and trans‐oleic acid (C18:1T) were detected exclusively in tucumã, further emphasizing the uniqueness of its lipid composition.

In contrast, uxi presented a markedly different fatty acid profile, characterized by the predominance of cis‐oleic acid (C18:1C), which accounted for 58.85 ± 0.67% of the total fatty acids. This was followed by palmitic acid (18.32 ± 0.14%), stearic acid (8.21 ± 0.13%), cis‐linoleic acid (C18:2C; 6.24 ± 0.15%), and α‐linolenic acid (C18:3; 3.55 ± 0.14%). Cis‐linoleic acid, α‐linolenic acid, docosadienoic acid (C22:2), heptadecanoic acid (C17:0), arachidic acid (C20:0), eicosenoic acid (C20:1), and eicosadienoic acid (C20:2) were all significantly more abundant in uxi following FDR correction (*q* < 0.01). Moreover, cis‐oleic acid, the predominant fatty acid in uxi, was not detected in tucumã.

Interestingly, not all fatty acids differ between species. Myristic acid (C14:0) and lignoceric acid (C24:0) showed no significant differences after FDR correction, indicating that these saturated fatty acids remain relatively conserved despite the pronounced divergence observed for most of the lipid fraction.

The predominance of cis‐oleic acid in uxi is particularly relevant from both nutritional and technological perspectives. Oleic acid is one of the principal monounsaturated fatty acids in edible vegetable oils and has been extensively associated with improved cardiovascular health, partly through its ability to reduce low‐density lipoprotein (LDL) cholesterol while maintaining or increasing high‐density lipoprotein (HDL) cholesterol (Zou and Liu 2025; Hua et al. 2026). From a technological perspective, the predominance of monounsaturated fatty acids may favor greater resistance to oxidative deterioration compared with lipid profiles richer in polyunsaturated fatty acids, owing to their lower degree of unsaturation (Gharby et al. 2025; Lankanayaka et al. 2025). However, this interpretation remains hypothesis‐based in the context of the present study, since the intrinsic oxidative stability of the lipid fractions from tucumã and uxi was not directly evaluated using accelerated oxidation methods such as the Rancimat test. Therefore, the potential relationship between the fatty acid profiles and oxidative stability should be confirmed through direct oxidation studies.

Conversely, tucumã was characterized by a substantially higher proportion of polyunsaturated fatty acids, particularly linoleic acid derivatives. Polyunsaturated fatty acids are nutritionally valuable because they participate in membrane structure, cellular signaling, and immune regulation; however, their multiple double bonds make them more susceptible to lipid oxidation (Dyall et al. 2022; Mortensen et al. 2023). Interestingly, despite this apparent susceptibility, tucumã exhibited elevated concentrations of phenolic compounds and flavonoids, suggesting that its endogenous antioxidant constituents may contribute to protecting the lipid fraction against oxidative degradation.

The presence of essential fatty acids further enhances the nutritional value of both fruits. Uxi contained significantly higher concentrations of cis‐linoleic acid (ω‐6) and α‐linolenic acid (ω‐3), both recognized for their roles in maintaining cell membrane integrity, regulating inflammatory responses, and reducing the risk of chronic non‐communicable diseases (Mititelu et al. 2024; Berkowitz et al. 2026). Together, these compounds reinforce the functional value of uxi as a source of health‐promoting lipids.

Overall, the statistically distinct fatty acid profiles demonstrate complementary nutritional, physiological, and potential technological characteristics between the two Amazonian fruits. Tucumã represents an important source of polyunsaturated fatty acids associated with bioactive phytochemicals, which may contribute to membrane integrity, cellular signaling, and immune regulation (Dyall et al. 2022; Mortensen et al. 2023). However, the higher degree of unsaturation of these fatty acids is generally associated with greater susceptibility to oxidative degradation, suggesting that the endogenous antioxidant compounds naturally present in tucumã may play an important role in protecting its lipid fraction. In contrast, uxi is distinguished by its high oleic acid content, which is associated with recognized cardiometabolic benefits and, based on the lower susceptibility of monounsaturated fatty acids to lipid oxidation, may favor greater oxidative resistance (Zou and Liu 2025; Gharby et al. 2025). Consequently, these distinct lipid profiles may influence the potential applicability of each fruit in different food systems, with uxi potentially representing a suitable ingredient for lipid‐rich products in which oxidative stability is relevant, whereas tucumã combines a nutritionally valuable polyunsaturated fatty acid profile with a rich antioxidant phytochemical composition that may help counterbalance the greater oxidative susceptibility associated with its lipid fraction (Jardim et al. 2024). However, the relationship between these fatty acid profiles and the intrinsic oxidative stability of the fruits remains hypothetical in the context of the present study, since direct oxidation measurements, such as the Rancimat test, were not performed. Future studies employing direct oxidative stability assays are therefore required to confirm whether the oleic acid‐rich profile of uxi effectively translates into greater resistance to lipid oxidation. These complementary lipid characteristics, together with the antioxidant properties demonstrated in the subsequent sections, reinforce the potential application of both fruits as functional food ingredients and promising natural sources of antioxidants for food preservation (Jardim et al. 2024; Shahidi and Samarasinghe 2025).

### 3.3 HPLC‐DAD Characterization

**FIGURE 1:**
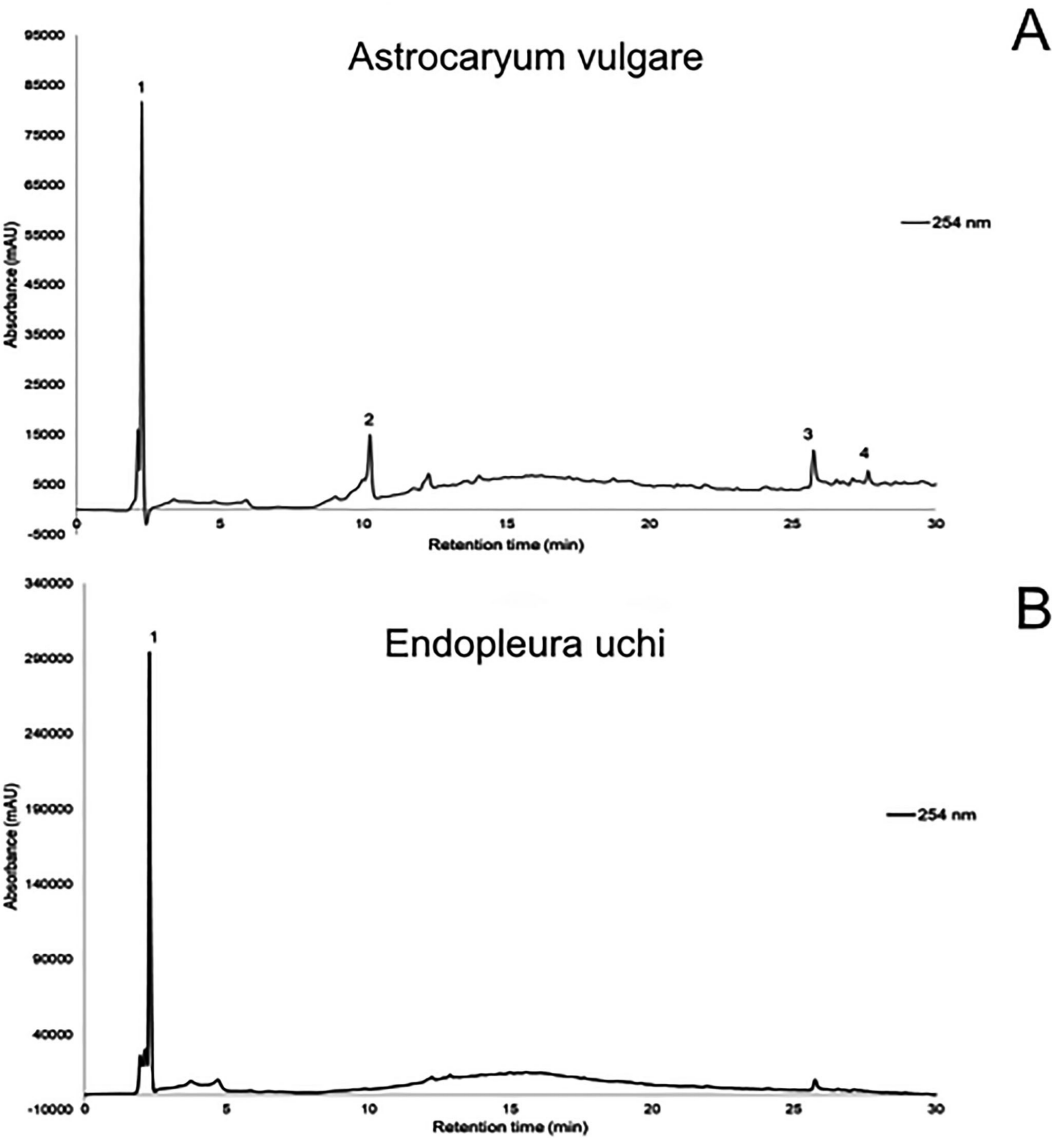
Representative HPLC‐DAD chromatograms (254 nm) of hydroalcoholic extracts from (A) *Astrocaryum vulgare* (tucumã) and (B) *Endopleura uchi* (uxi). Numbered peaks correspond to the compounds tentatively identified in Table 2. Peak identities are as follows: (1) citric acid (tucumã) or gallic acid (uxi), (2) gallic acid, (3) quercetin derivative, and (4) quercetin derivative. Chromatographic conditions are described in Section 2.5.

The chromatographic profiles of the hydroalcoholic extracts of tucumã (*A. vulgare*) and uxi (*E. uchi*) obtained by HPLC‐DAD at 254 nm are presented in Figure 1, while the tentative identification of the major compounds is summarized in Table 2. Distinct chromatographic patterns were observed between the two Amazonian fruits, indicating substantial differences in their phytochemical composition.

**TABLE 2:** Tentative identification of the major compounds detected in the hydroalcoholic extracts of *Astrocaryum vulgare* and *Endopleura uchi* by HPLC‐DAD.

Plant species/standards	Peak no.	RT (min)	*λ*_máx_ (nm)	Peak area (mAU·s)	Quantification (mg eq./L)	Tentatively identified compound	Chemical class
*Astrocaryum vulgare*	1	2.283	271	548,027	163.4986631	Citric acid	Organic acid
*Astrocaryum vulgare*	2	10.247	273	298,430	13.06262958	Gallic acid	C3–C6 phenolic acid
*Astrocaryum vulgare*	3	25.739	253	97,296	2.922173098	Quercetin	Glycosylated flavonol
*Astrocaryum vulgare*	4	27.645	257	22,034	1.627432091	Quercetin	Glycosylated flavonol
*Endopleura uxi*	1	2.281	287	1,486,071	59.52200446	Gallic acid	Simple phenolic acid
Citric acid	Standard	2.286	271			Citric acid	Organic acid
Gallic acid	Standard	3.398	271			Gallic acid	Simple phenolic acid
Rutin	Standard	14.584	256			Rutin	Glycosylated flavonol
Quercetin	Standard	18.984	256			Quercetin	Flavonol aglycone

*Note*: Retention time (RT), maximum absorption wavelength (*λ*_max_), chromatographic peak area, estimated concentration, tentative compound identification, and chemical classification of the major compounds detected in the hydroalcoholic extracts of tucumã (*Astrocaryum vulgare*) and uxi (*Endopleura uchi*). Compound identification was based on comparison of retention times and UV–vis absorption spectra with authentic analytical standards and published literature.

The chromatogram of tucumã exhibited a more complex profile, characterized by four major peaks (Figure 1A). Peak 1, detected at a RT of 2.283 min, represented the predominant compound in the extract, accounting for approximately 57% of the total chromatographic area and presenting a concentration of 163.50 mg eq. L^−1^. Based on RT and UV absorption spectra, this compound was tentatively identified as citric acid (Table 2). The predominance of citric acid is consistent with the organic acid composition commonly reported for tropical fruits and may contribute to both flavor characteristics and oxidative stability (Sorathiya et al. 2025).

Peak 2 (RT = 10.247 min) was tentatively identified as gallic acid, a phenolic acid widely recognized for its antioxidant properties. Gallic acid and its derivatives are frequently associated with radical scavenging activity and protection against oxidative damage in food systems (Molski 2023; Hadidi et al. 2024). The presence of this compound is consistent with the elevated TPC observed for tucumã.

Peaks 3 and 4, detected at RTs of 25.739 and 27.645 min, respectively, exhibited UV absorption spectra characteristic of flavonol derivatives and were tentatively identified as quercetin‐related glycosides (Table 2). Although present at lower concentrations, these compounds may contribute significantly to the antioxidant activity of the extract due to their well‐established ability to scavenge reactive oxygen species and inhibit oxidative chain reactions (Tumilaar et al. 2024).

In contrast, the chromatographic profile of uxi was considerably simpler (Figure 1B), being dominated by a single major peak detected at RT = 2.281 min. This compound exhibited a maximum absorption wavelength of 287 nm and was tentatively identified as gallic acid, representing the major phenolic constituent detected under the chromatographic conditions employed. The large chromatographic area associated with this peak suggests that antioxidant activity in uxi may be largely attributed to a limited number of highly abundant phenolic compounds.

The distinct chromatographic profiles observed between tucumã and uxi help explain the differences in total phenolic and flavonoid contents reported in the present study. While tucumã displayed greater phytochemical diversity, including organic acids, phenolic acids, and flavonol derivatives, uxi exhibited a less complex profile dominated by a single phenolic constituent. Despite these differences, both fruits contained compounds with recognized antioxidant properties, supporting their potential application as natural ingredients for food preservation.

Overall, the HPLC‐DAD analysis confirmed the presence of bioactive metabolites associated with antioxidant activity, including citric acid, gallic acid, and quercetin derivatives. These findings provide a chemical basis for the antioxidant responses observed in the subsequent in vitro assays and reinforce the technological potential of these Amazonian fruits as natural food preservatives. However, it should be noted that the identification of these compounds was considered tentative, as it was based on RTs and UV–visible absorption spectra obtained by HPLC‐DAD. Likewise, the concentrations reported in Table 2 should be interpreted as semi‐quantitative estimates based on chromatographic response and comparison with the corresponding standards, rather than as absolute concentrations of the compounds in the extracts. Therefore, comparisons between tucumã and uxi refer to estimated relative differences under the analytical conditions employed. Future studies employing liquid chromatography coupled with mass spectrometry (LC‐MS/MS) and validated compound‐specific quantitative methods are warranted to confirm the identity and absolute concentrations of the detected compounds and provide a more comprehensive characterization of the phytochemical composition of tucumã and uxi.

### 3.4 Antioxidant Activity

**FIGURE 2:**
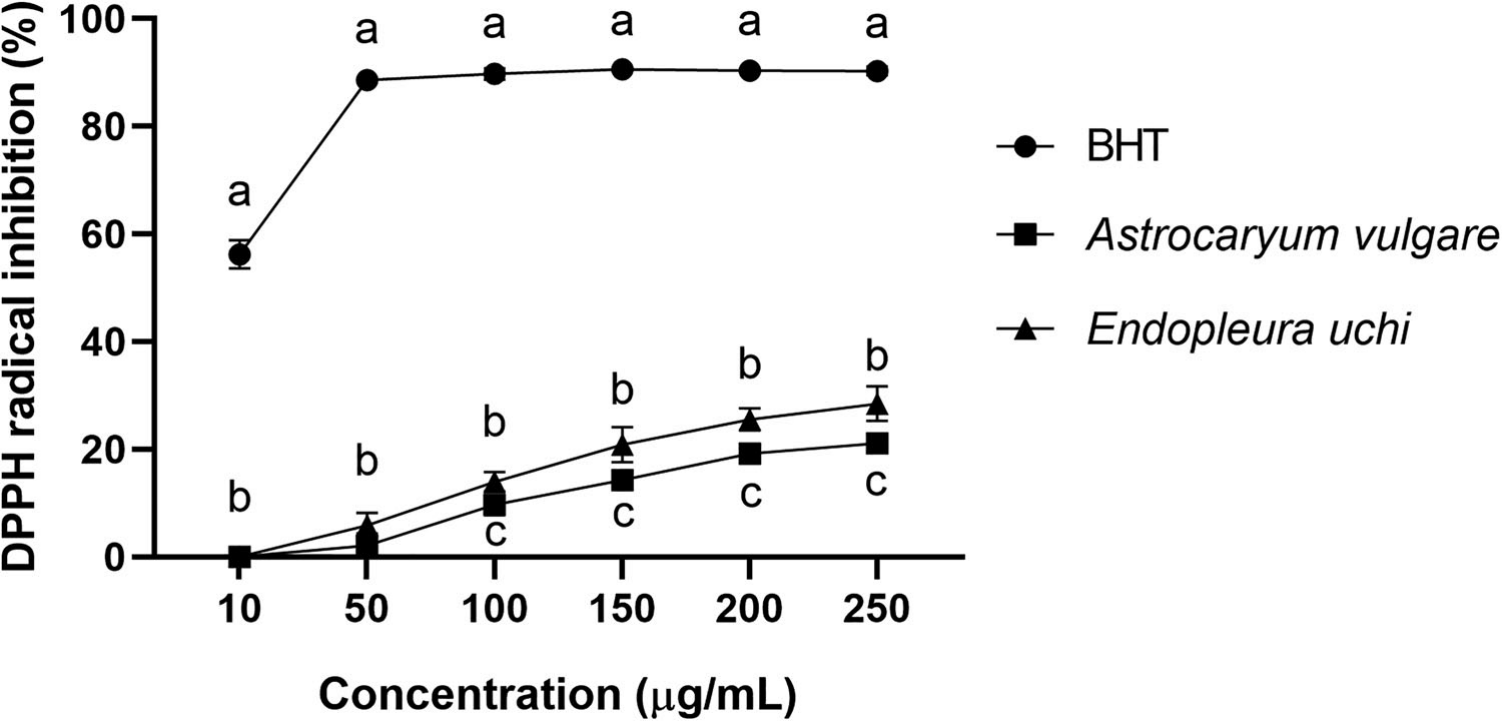
DPPH radical scavenging activity of BHT, *Astrocaryum vulgare*, and *Endopleura uchi* extracts at different concentrations. Data are presented as mean ± SD (*n* = 3). Different lowercase letters indicate significant differences among samples within the same concentration according to two‐way ANOVA followed by Šídák's multiple comparisons test (*p* < 0.05).

The antioxidant potential of *A. vulgare* (tucumã) and *E. uchi* (uxi) extracts was investigated using complementary assays based on distinct antioxidant mechanisms, including FRAP, TAC, and DPPH radical scavenging activity (Figure 2).

The FRAP assay revealed significant differences between the two Amazonian fruits. Uxi exhibited a significantly greater ferric ion reducing capacity (0.41 ± 0.08 mmol Fe^2+^/g extract) than tucumã (0.18 ± 0.04 mmol Fe^2+^/g extract) (Welch's *t*‐test, *p* = 0.0191), demonstrating a superior electron‐donating ability. This result is particularly noteworthy because tucumã contained significantly higher total phenolic and flavonoid contents, indicating that antioxidant efficiency depends not only on the overall abundance of phytochemicals but also on their chemical composition and intrinsic redox properties (Gulcin 2025; Gualberto et al. 2025 Shahidi and Samarasinghe 2025). In agreement with the HPLC‐DAD analysis, the predominance of gallic acid in uxi may partially explain its greater reducing capacity. Although tucumã exhibited significantly higher total phenolic and flavonoid contents, antioxidant activity depends not only on the abundance of phenolic compounds but also on their molecular structure, intrinsic redox properties, and possible synergistic interactions within the phytochemical matrix (Gulcin 2025; Shahidi and Samarasinghe 2025). Gallic acid is recognized for its remarkable electron‐transfer ability due to the presence of three hydroxyl groups attached to the aromatic ring, making it particularly effective in electron‐transfer‐based assays such as FRAP (Wianowska and Olszowy‐Tomczyk 2023; Hadidi et al. 2024). Notably, the estimated concentration of gallic acid in uxi was approximately 4.6‐fold higher than that observed in tucumã, which is consistent with the superior FRAP and DPPH activities exhibited by uxi. However, because the present study evaluated only two fruit species, this relationship should be interpreted as a qualitative association rather than a statistically validated correlation. Since the FRAP assay is based exclusively on single‐electron transfer reactions, these findings suggest that the stronger reducing activity of uxi reflects qualitative differences in phytochemical composition rather than simply a higher abundance of total phenolics, which may contribute to enhanced oxidative stability in food systems (Nwachukwu et al. 2021).

In contrast, the phosphomolybdenum assay showed no significant difference in TAC between the two fruits (Welch's unpaired *t*‐test: *t* = 0.027, df = 3.98, *p* = 0.980). Tucumã and uxi presented TAC values of 0.592 ± 0.102 and 0.589 ± 0.110 g ascorbic acid equivalents (AAE)/g extract, respectively. Although uxi displayed superior reducing power in the FRAP assay, both extracts exhibited comparable TAC when evaluated by a method that integrates multiple electron‐transfer reactions. This result highlights the importance of employing complementary antioxidant assays, since different classes of bioactive compounds contribute differently depending on the reaction mechanism being assessed (Nwachukwu et al. 2021; Shahidi and Samarasinghe 2025). Consequently, the combined use of multiple antioxidant assays provides a more comprehensive evaluation of the antioxidant potential of complex plant matrices than any single analytical method (Nwachukwu et al. 2021; Zheng et al. 2024; Shahidi and Samarasinghe 2025).

The DPPH radical scavenging assay further demonstrated that the antioxidant activity of both extracts was concentration‐dependent (Figure 2). Two‐way ANOVA revealed significant effects of sample type (*F*(2,36) = 7671, *p* < 0.0001), extract concentration (*F*(5,36) = 267.7, *p* < 0.0001), and a significant interaction between these factors (*F*(10,36) = 23.01, *p* < 0.0001), indicating that the antioxidant response varied according to both botanical source and concentration. As expected, the synthetic antioxidant BHT exhibited significantly greater DPPH radical scavenging activity than both fruit extracts at all evaluated concentrations (Sidak's multiple comparisons, *p* < 0.0001). No significant differences between tucumã and uxi were observed at 10 and 50 mg/L (*p* = 0.9150 and 0.0797, respectively). However, beginning at 100 mg/L, uxi exhibited significantly greater radical scavenging activity than tucumã (*p* = 0.0354), with the difference becoming more pronounced at 150 mg/L (*p* = 0.0008), 200 mg/L (*p* = 0.0012), and 250 mg/L (*p* = 0.0002). At the highest concentration evaluated, DPPH inhibition reached approximately 29.6% for uxi and 21.2% for tucumã, demonstrating a stronger concentration‐dependent antioxidant response for the uxi extract.

The concentration‐response profile was further summarized by the EC_50_ values. As expected, BHT presented the lowest EC_50_ (0.051 ± 0.011 mg extract/mg DPPH), differing significantly from both fruit extracts (one‐way ANOVA followed by Tukey's test, *p* < 0.0001). Uxi exhibited a numerically lower EC_50_ (18.03 ± 1.12 mg extract/mg DPPH) than tucumã (20.91 ± 2.36 mg extract/mg DPPH), indicating that a lower amount of extract was required to achieve 50% radical scavenging. Nevertheless, this difference was not statistically significant (*p* = 0.1238). It should be noted, however, that the EC_50_ values were estimated from the concentration range evaluated in the present study and should therefore be interpreted as approximate indicators of antioxidant potency rather than absolute parameters. Although this concentration range was sufficient to discriminate the antioxidant performance of the two fruits, broader concentration intervals could provide more robust EC_50_ estimates and facilitate comparisons with the literature. Accordingly, future work is planned to extend the concentration range evaluated for both extracts, allowing the construction of more comprehensive dose‐response curves and more robust EC_50_ estimation. Complementary antioxidant metrics, such as Trolox equivalent antioxidant capacity (TEAC), will also be considered to improve standardization and facilitate comparisons across studies.

Taken together, the results obtained from FRAP, TAC, and DPPH demonstrate that the antioxidant properties of these Amazonian fruits are complementary rather than identical. Whereas uxi exhibited greater reducing power and superior radical scavenging activity at higher concentrations, tucumã showed higher total phenolic and flavonoid contents. The absence of differences in TAC, combined with the superior performance of uxi in FRAP and DPPH assays, reinforces that antioxidant activity is governed primarily by the qualitative composition and reactivity of phytochemicals rather than by their total concentration alone, highlighting the importance of evaluating complementary antioxidant mechanisms to characterize complex plant extracts (Zheng et al. 2024; Shahidi and Samarasinghe 2025; Brito et al. 2026). These complementary antioxidant profiles suggest that tucumã and uxi may fulfill distinct yet synergistic roles in food preservation. While uxi appears particularly suitable for applications requiring greater reducing power and radical scavenging capacity, tucumã may provide broader phytochemical diversity through its higher phenolic and flavonoid contents. Together, these characteristics reinforce the potential application of both fruits as natural antioxidant ingredients for food preservation. In addition to conventional food products, such antioxidant‐rich extracts may also represent promising natural alternatives for post‐harvest preservation of highly perishable foods, including edible mushrooms, where oxidative deterioration is a major factor affecting quality and shelf life (Zheng et al. 2025).

### 3.5 Inhibition of Lipid Peroxidation (TBARS Assay)

**FIGURE 3:**
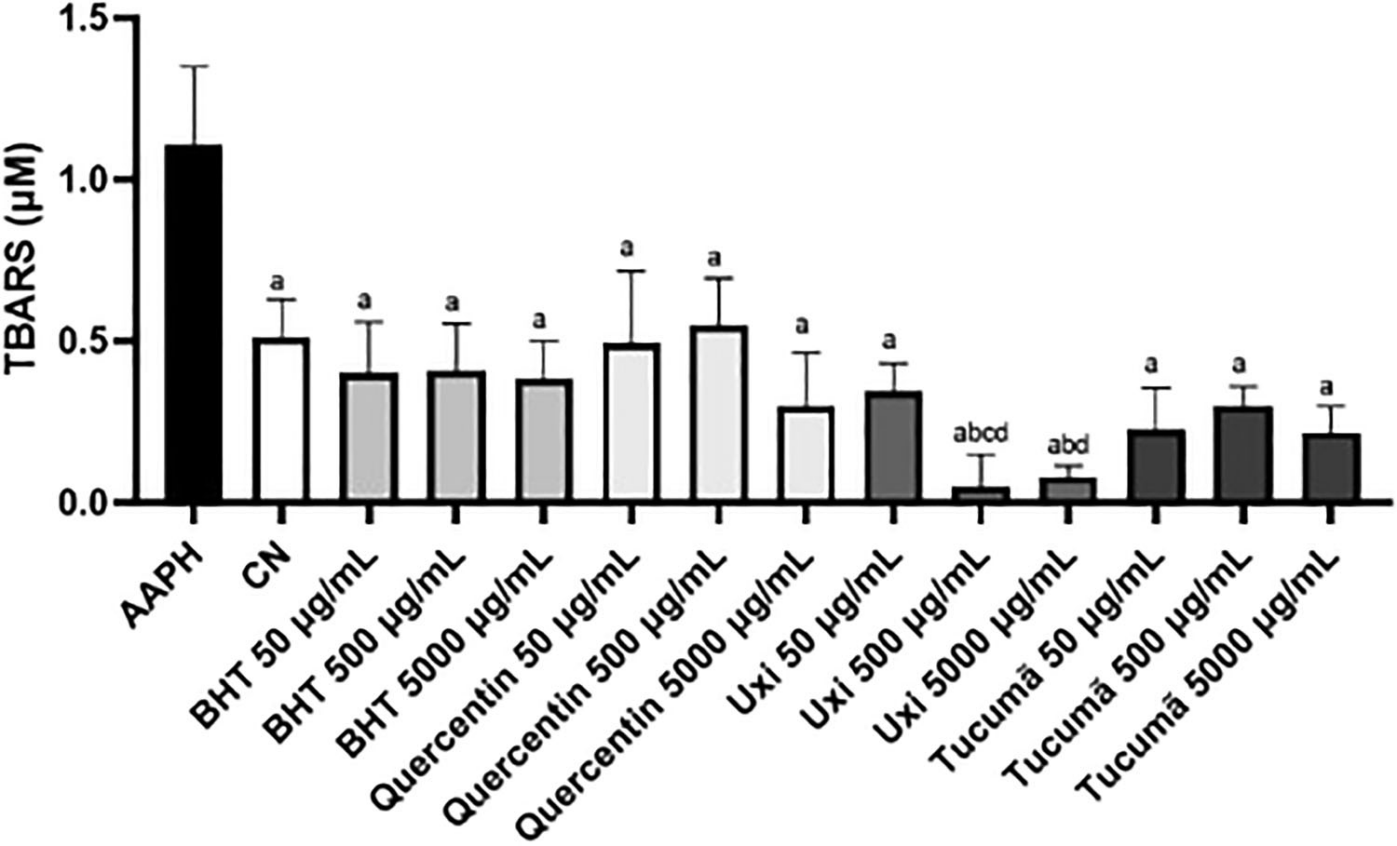
Inhibitory effect of hydroalcoholic extracts of *Astrocaryum vulgare* (tucumã) and *Endopleura uchi* (uxi) on AAPH‐induced lipid peroxidation, determined by the thiobarbituric acid reactive substances (TBARS) assay. BHT and quercetin were used as reference antioxidants, and the negative control (CN) consisted of AAPH‐treated samples without antioxidants. Data are presented as mean ± SD (*n* = 5). Different lowercase letters indicate significant differences among treatments according to one‐way ANOVA followed by Tukey's multiple comparisons test (*p* < 0.05).

The capacity of tucumã (*A. vulgare*) and uxi (*E. uchi*) extracts to inhibit lipid peroxidation was evaluated using the TBARS assay, and the results are presented in Figure 3. Lipid oxidation induced by AAPH resulted in the highest TBARS levels, confirming the effectiveness of the oxidative challenge and establishing a suitable model for evaluating the protective effects of the extracts.

Both fruit extracts reduced TBARS formation compared with the oxidized control, demonstrating their ability to protect lipid substrates against oxidative damage. However, the magnitude of this protection differed between species and concentrations. Uxi exhibited the strongest inhibitory effect, particularly at 500 and 5000 µg/mL, where TBARS levels were markedly reduced and statistically different from the oxidized control. These findings indicate a concentration‐dependent protective effect and suggest that compounds present in uxi are highly effective at preventing the propagation of lipid peroxidation reactions.

Tucumã also decreased lipid oxidation at all evaluated concentrations, although its effect was less pronounced than that observed for uxi. Nevertheless, the reduction in TBARS formation confirms that the antioxidant compounds present in tucumã remain active in lipid‐rich systems and contribute to the stabilization of oxidizable substrates.

The strong inhibitory activity observed for uxi is particularly noteworthy considering its chromatographic profile, which was dominated by gallic acid‐related compounds. Phenolic acids are known to interrupt free‐radical chain reactions, donate hydrogen atoms, and stabilize reactive intermediates generated during lipid oxidation (Platzer et al. 2022; Chaudhary et al. 2023; Tumilaar et al. 2024). Likewise, the presence of flavonoids and other phenolic constituents in tucumã may explain its protective activity despite its lower effectiveness in this assay (Machado et al. 2022; Özdemir et al. 2026).

The ability to inhibit lipid peroxidation represents one of the most relevant characteristics of natural food preservatives, since oxidative deterioration is a major cause of quality loss in lipid‐containing foods (Félix et al. 2020; Eze et al. 2025). Oxidation can negatively affect flavor, aroma, color, nutritional value, and shelf life (Araújo et al. 2023; Gharby et al. 2025). Although the egg yolk TBARS assay is a well‐established model for assessing lipid peroxidation, it represents a simplified experimental system and does not fully reproduce the physicochemical complexity of real food matrices. Therefore, the reduction of TBARS formation observed for both Amazonian fruits should be interpreted as preliminary evidence supporting their potential application as natural antioxidant ingredients for food preservation, which should be further validated in representative food systems.

Taken together, the results of the TBARS assay corroborate the findings obtained in the phytochemical characterization and antioxidant assays, demonstrating that the bioactive compounds present in tucumã and uxi can exert protective effects in complex lipid systems. Among the evaluated fruits, uxi showed the greatest potential for preventing lipid oxidation, consistent with its superior performance in the FRAP and DPPH assays. Although the effective concentrations evaluated in this study were higher than those commonly reported for purified synthetic antioxidants, direct comparisons should be interpreted with caution because the hydroalcoholic extracts represent complex mixtures of naturally occurring bioactive compounds rather than isolated antioxidant molecules. From a practical perspective, particularly the highest concentration tested (5000 µg/mL) may not be directly transferable to food products, since incorporation of hydroalcoholic fruit extracts at relatively high concentrations could potentially affect sensory attributes such as color, flavor, aroma, or texture, depending on the characteristics of the food matrix. Because sensory properties were not evaluated in the present study, the concentration range capable of providing adequate oxidative protection without compromising product acceptability remains to be established. Accordingly, the next step will be to evaluate tucumã and uxi extracts in actual lipid‐rich food matrices, including edible oils, emulsified systems, and meat products, to determine their minimum effective concentrations, antioxidant efficacy, impact on oxidative stability, and sensory acceptability under realistic processing and storage conditions. Sensory testing will be particularly important to determine whether the incorporation of these extracts at technologically effective concentrations is compatible with consumer acceptance and their potential use in clean‐label food formulations.

## 4 Conclusions

This study demonstrated that the Amazonian fruits tucumã (*A. vulgare*) and uxi (*E. uchi*) possess distinct phytochemical compositions and complementary antioxidant properties, highlighting their potential as natural ingredients for food preservation. Although tucumã contained significantly higher concentrations of total phenolics and flavonoids, uxi exhibited greater ferric reducing power, superior DPPH radical scavenging activity at higher concentrations, and stronger inhibition of lipid peroxidation. In contrast, both fruits showed comparable TAC, indicating that different antioxidant mechanisms contribute to their overall protective effects.

The integration of phytochemical characterization, fatty acid profiling, HPLC‐DAD analysis, and multiple antioxidant assays demonstrated that antioxidant performance is determined primarily by the qualitative composition of bioactive compounds rather than by the total concentration of phenolics alone. In particular, the predominance of gallic acid in uxi may explain its superior reducing capacity despite its lower phenolic content, emphasizing the importance of compound‐specific composition in predicting antioxidant functionality.

Overall, these findings identify tucumã and uxi as complementary sources of natural antioxidants with promising applications as clean‐label ingredients for improving the oxidative stability of food products. Furthermore, this study contributes to the valorization of Amazonian biodiversity by providing scientific evidence supporting the technological use of these underexplored fruits in the development of safer and more sustainable food preservation strategies.

## Author Contributions

**Naikita Suellen da Silva e Silva**: conceptualization, methodology, investigation, formal analysis, data curation, visualization, writing – original draft. **Thaís Oliveira Freitas**: investigation, formal analysis, data curation. **Ana Paula Barros Soares**: investigation, formal analysis. **Bruna Sumaya Dias Pinheiro**: investigation, formal analysis. **Saulo Victor e Silva**: writing – review and editing. **Anelise Christ‐Ribeiro**: investigation, formal analysis. **Jefferson Romáryo Duarte da Luz**: methodology, supervision, writing – review and editing. **Gabriel Araujo‐Silva**: conceptualization, methodology, supervision, resources, funding acquisition, writing – review and editing.

## Funding

This work was supported by the Fundo JBS pela Amazônia. Naikita Suellen da Silva e Silva received a scholarship from the Coordenação de Aperfeiçoamento de Pessoal de Nível Superior (CAPES), Brazil (Finance code: 001), which also supported the article processing charge (APC). Gabriel Araujo‐Silva acknowledges financial support from the Fundação de Amparo à Pesquisa do Estado do Amapá (FAPEAP) and the Conselho Nacional de Desenvolvimento Científico e Tecnológico (CNPq) through the State Productivity Fellowship Program (Public Call No. 18/2024, Grant No. 051/2025).

## Conflicts of Interest

The authors declare no conflicts of interest.

## Data Availability

The data supporting the findings of this study are available from the corresponding author upon reasonable request.
